# Immune related adverse events associated with anti-CTLA-4 antibodies: systematic review and meta-analysis

**DOI:** 10.1186/s12916-015-0455-8

**Published:** 2015-09-04

**Authors:** Anne Bertrand, Marie Kostine, Thomas Barnetche, Marie-Elise Truchetet, Thierry Schaeverbeke

**Affiliations:** Département de Rhumatologie, Hôpital Pellegrin, CHU de Bordeaux, Bordeaux, France; Laboratoire d’Immunologie, UMR-CNRS 5164, Université de Bordeaux, Bordeaux, France; Unité sous Contrat, Infections à Mycoplasmes et à Chlamydia chez l’Homme, Université de Bordeaux, Bordeaux, France

**Keywords:** Anti-CTLA4 antibodies, Colitis, Dermatitis, Hypohysitis, Immune related adverse events, Ipilimumab, Metastatic tumors, Oncology, Tremelimumab

## Abstract

**Background:**

Targeting CTLA-4 is a recent strategic approach in cancer control: blocking CTLA-4 enhances an antitumor immunity by promoting T-cell activation and cytotoxic T-lymphocyte proliferation. This induction of a tolerance break against the tumor may be responsible for immune-related adverse events (irAEs). Our objective was to assess the incidence and nature of irAEs in oncologic patients receiving anti-CTLA-4 antibodies (ipilimumab and tremelimumab).

**Methods:**

A systematic search of literature up to February 2014 was performed in MEDLINE, EMBASE, and Cochrane databases to identify relevant articles. Paired reviewers independently selected articles for inclusion and extracted data. Pooled incidence was calculated using R^©^, package meta.

**Results:**

Overall, 81 articles were included in the study, with a total of 1265 patients from 22 clinical trials included in the meta-analysis. Described irAEs consisted of skin lesions (rash, pruritus, and vitiligo), colitis, and less frequently hepatitis, hypophysitis, thyroiditis, and some rare events such as sarcoidosis, uveitis, Guillain-Barré syndrome, immune-mediated cytopenia and polymyalgia rheumatic/Horton. The overall incidence of all-grade irAEs was 72 % (95 % CI, 65–79 %). The overall incidence of high-grade irAEs was 24 % (95 % CI, 18–30 %). The risk of developing irAEs was dependent of dosage, with incidence of all-grade irAEs being evaluated to 61 % (95 % CI, 56–66 %) for ipilimumab 3 mg/kg and 79 % (95 % CI, 69–89 %) for ipilimumab 10 mg/kg. Death due to irAEs occurred in 0.86 % of patients.

The median time of onset of irAEs was about 10 weeks (IQR, 6–12) after the onset of treatment, corresponding with the first three cycles but varied according to the organ system involved. Such immune activation could also be indicative for tumor-specific T-cell activation and irAE occurrence was associated with clinical response to CTLA-4 blocking in 60 % of patients.

**Conclusion:**

The price of potential long-term survival to metastatic tumors is an atypical immune toxicity, reflecting the mechanism of action of anti-CTLA-4 antibodies. A better knowledge of these irAEs and its management in a multidisciplinary approach will help to reduce morbidity and therapy interruptions.

**Electronic supplementary material:**

The online version of this article (doi:10.1186/s12916-015-0455-8) contains supplementary material, which is available to authorized users.

## Background

Approval of the first cancer immunotherapies suggests a promising therapeutic approach in some metastatic cancers [1]. In contrast to most oncologic treatments, antibodies target lymphocyte receptors or their ligands (and not the tumor cells directly) in order to enhance endogenous anti-tumor activity. Among these receptors, cytotoxic T-lymphocyte-associated antigen-4 (CTLA-4) is a particularly important immune checkpoint receptor and the first to be clinically targeted in oncology.

**Fig. 1:**
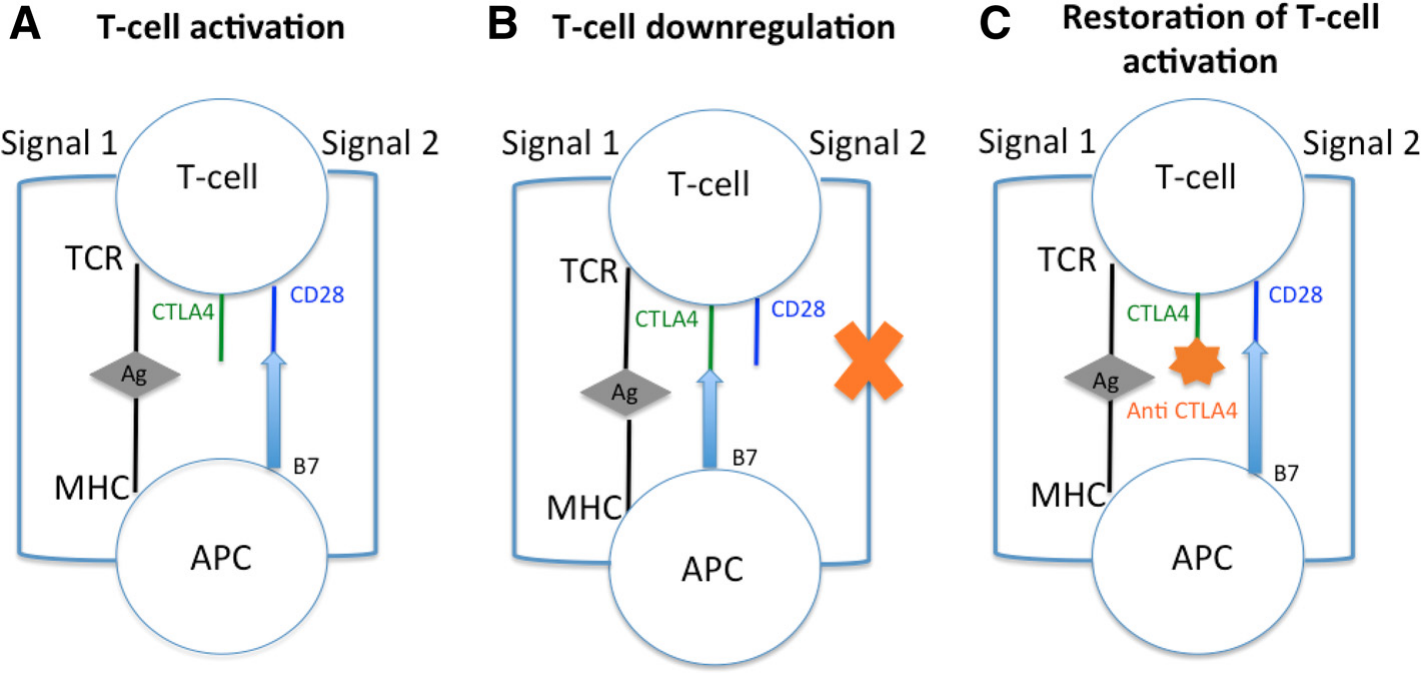
Mechanism of action: CTLA-4 and anti-CTLA-4 antibodies. Two signals are required to initiate an immune response. For the first signal (signal 1), tumor associated antigen (Ag), is presented by major histocompatibility complex (MHC) on antigen presenting cell (APC) and recognized by the toll-like receptor (TCR) of T-cell. Signal 2 occurs in response to binding of CD80 or CD86 (B7) on APC cell with CD28 receptor on T-cell (**a**). CTLA-4 is a homolog of CD28 and limits proliferative response of activated T-cell competing with CD28 for ligand B7. This inhibition occurs in response to binding of CD80 or CD86 on APC with CTLA-4 receptor on T-cell and interrupts signal 2 (**b**). Anti-CTLA-4 antibodies blocks CTLA-4 and enhances T-cell activation and proliferation (**c**)

The concept of immunosurveillance and involvement of the immune system in cancer development has been known for several years [2]. In the last two decades, efforts to activate anti-cancer host immunity were focused on T-cells due to their central role in the anti-cancer adaptive immune response, which is regulated by numerous co-stimulatory and inhibitory signals through tumor antigen recognition by the T-cell receptor. Thus, blockades of immune checkpoints with antagonists of inhibitory pathways have been developed and anti-CTLA-4 antibodies are precursors in this domain [3] (Mechanism of action: anti CTLA-4 antibodies in Fig. 1). CTLA-4, expressed exclusively on T-cells, acts as a negative co-stimulatory signal, inhibiting T-cell activation and proliferation to maintain self-tolerance and protect from autoimmunity [4]. This role is supported by the lethal lympho-proliferation and autoimmunity in CTLA-4 knockout mice [5]. Recently, heterozygous germline mutations in CTLA-4 have been identified in four unrelated families with severe immune dysregulation [6]. Interestingly, these mutations show the spectrum of clinical immune complications that can be anticipated from anti-CTLA-4 drugs.

Thus, a fully humanized anti-CTLA-4 monoclonal antibody immunoglobulin (Ig) G1 isotype (ipilimumab) was approved by the Food and Drug Administration (FDA) in 2011 at a dosage of 3 mg/kg every 3 weeks for four cycles in advanced melanoma, with evidence of improved survival [7, 8]. An IgG2 isotype (tremelimumab) was developed at a dosage of 15 mg/kg every 90 days to minimize complement activation and reduce the risk of cytokine release syndrome [9]. The clinical value of anti-CTLA-4 antibodies is being investigated in various cancer types including prostate, renal, bladder, colorectal, esophageal, pancreatic, gastric, hepatocellular, and pulmonary malignancies as well as mesothelioma and lymphoma [10].

As might be expected with blocking CTLA-4, the induction of a tolerance break against the tumor may be responsible for a variety of specific immune-related adverse events (irAEs) that occur in approximately 60 % of the patients treated by ipilimumab [7]. These include skin, gastrointestinal, hepatic, endocrine, neurologic, hematologic, ophthalmologic, and rheumatologic autoimmune diseases.

Our objective was to assess the incidence and the nature of irAEs in oncologic treatment with anti-CTLA-4 antibodies (ipilimumab and tremelimumab) through a systematic review and meta-analysis of the literature.

## Methods

### Data sources and searches

A systematic literature search was performed up to February 2014 in the Medline, Embase, and Cochrane databases to identify relevant articles. Two investigators (AB and MK) together determined the optimal search strategy checking several combinations of keywords with the Boolean logical operators AND/OR. A consensus was reached and validated by all authors. Hence, the keywords used were “safety OR security OR side effects OR adverse events AND (anti CTLA4 OR anti CTLA-4 OR ipilimumab OR tremelimumab)” in Medline and Embase, and “anti CTLA4 OR anti CTLA-4 OR ipilimumab OR tremelimumab” in the Cochrane database. The search was limited to studies on human beings and published in English. Manual searches from bibliographic references were also performed after reading of the first selection of articles. Articles published as full text were preferable in order to study irAEs in detail and to check the quality assessment of trials included in the meta-analysis. Additionally, many medical specialties are concerned with these side effects due to their diversity and it would thus be difficult to check all conference meetings in 2 years prior to the study. Therefore, unpublished studies were not searched.

### Eligibility criteria and study selection

We included clinical trials and case reports that reported irAEs in oncologic patients receiving anti-CTLA-4 antibodies (ipilimumab or tremelimumab). Patients included were adults with a diagnosis of metastatic cancer or unresectable tumor. They could have received previous oncologic therapy before inclusion. Patients with anti-CTLA-4 prescribed in combination with other treatments or patients with previous receipt of anti-CTLA-4 antibodies were excluded. The two investigators conducted study selection and manual searches in bibliographic references independently, selecting the relevant articles initially on the basis of titles and abstracts, and then on the full texts.

### Outcomes

Incidence evaluation was based on the number of irAEs for global and specific irAEs (skin, gastrointestinal, endocrine, and hepatic diseases) and their grade (1–5; recorded according to Version 2, 3 or 4 of the Common Terminology Criteria for Adverse Events of the National Cancer Institute). Grades ≥3 were considered high-grade.

### Data extraction

Each investigator performed the reading and data extraction independently. They used, for each study, a standard data extraction form and re-read together the articles in the event of any discrepancy in their interpretation. When data were not available, efforts were made to contact first authors.

Clinical trials were used to determine the incidence of irAEs. Information on the author and year of publication, population size, study design, treatment (ipilimumab or tremelimumab) and dosing regimen, duration of treatment (and number of infusions), and irAEs outcomes was extracted.

Case reports were used to describe the diversity of irAEs. Patient characteristics, the previous oncologic treatment, and the nature of each irAE, their onset, their treatment, and outcome were all recorded. Cancer outcome was also noted when reported in the article (partial or complete remission, stability, or progression).

### Quality assessment

The Cochrane Collaboration’s tool was used to assess risk of bias and to evaluate the quality of articles included in the meta-analysis [11], addressing sequence generation, allocation concealment, blinding of participants and personnel, incomplete outcome data, selective outcome reporting, and other sources of bias. Disagreements among investigators were discussed and agreement was reached by consensus.

### Data synthesis and analysis

The primary objective of the study was the number of irAEs for each group of treatment (ipilimumab and tremelimumab). An incidence was estimated for each study included in the meta-analysis estimation. Statistical heterogeneity among the selected studies was tested based on the Q-test (χ^2^), using a significant level of 0.05, and reported with the I^2^ statistic in which high values indicate high heterogeneity. If heterogeneity was not rejected by the Q-test or under a threshold of 30 %, all meta-analyses were carried out using the inverse variance approach (fixed-effect model); otherwise, the inverse variance corrected by the inter-study variability was used (random-effect model). This method was not relevant for the question addressed herein due to the presence of zero values in adverse event incidences. In this case, a correction (λ) was introduced in the variance calculation (classically adding 0.5 to the number of event counts), and the exact formula of variance became:

$$ \frac{\left( count+\lambda \right)\left(n- count+\lambda \right)}{{\left(n+2\lambda \right)}^3} $$

It provided a common weighted incidence estimate with 95 % confidence interval (CI), taking into account the weight of the different samples. Incidences and their 95 % CIs were shown on forest plots. Publication bias was assessed using the funnel plot method. All computations were performed using R software (R version 2.12.2 (2012-10-26) with the package Meta and the function Metaprop.

## Results

### Literature search

**Fig. 2:**
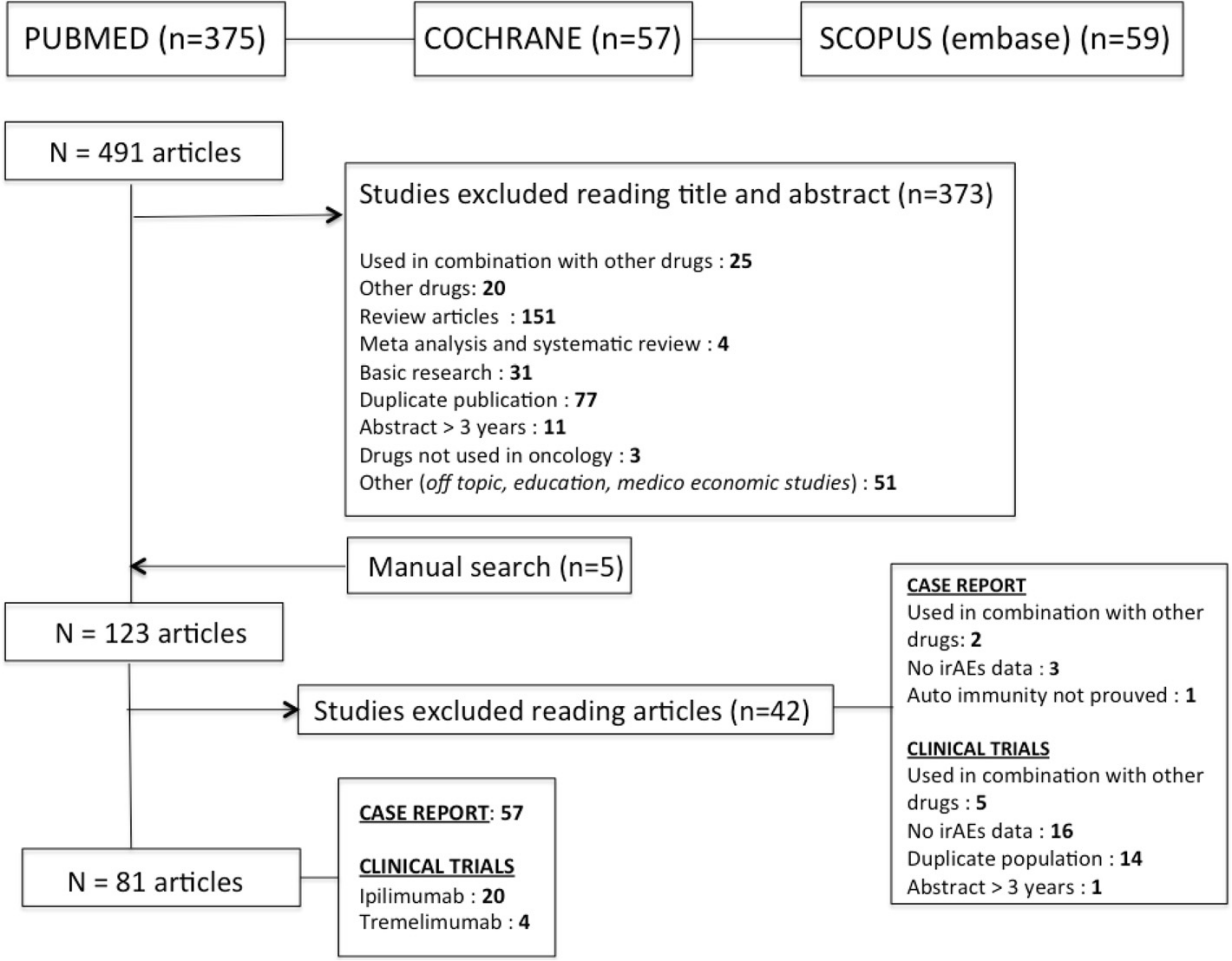
Flow diagram for identification and selection of studies included in the systematic review and meta-analysis

The literature search identified 491 articles in databases and manual searches retrieved five additional articles. Among these 496 articles, 373 were excluded after reading of the abstracts due to duplicate articles, oncologic treatment in combination with other drugs, review articles, or basic research. Finally, 123 articles were fully reviewed and 81 were considered relevant for the present study: 24 clinical trials and 57 case reports (Fig. 2).

Clinical trials in which patients were treated according to the labeling of the products (ipilimumab 3 mg/kg every 3 weeks for at least four cycles and tremelimumab at 15 mg/kg every 90 days) or at upper dosage were included for meta-analysis. As one study was retrospective and one used ipilimumab for only one or two doses, these two studies were only selected in the systematic review but not for meta-analysis [12, 13].

### Incidence of irAEs – data from clinical trials

#### General characteristics

In total, 1265 patients from 22 clinical trials were included for meta-analysis to assess the incidence of irAEs with anti-CTLA-4 treatment (Table 1) [7, 14–34]; 18 studies concerned ipilimumab treatment and four concerned tremelimumab, but incidence data of global irAEs for tremelimumab was available in only one study [31]. Moreover, the tremelimumab phase III randomized clinical trial in advanced melanoma was not included because 46 patients (14 %) received ipilimumab in the control arm [35]. Most of the studies were not blind (18/22). Single arm studies were retrieved in 11 articles, six studies were randomized and five were not. Half of the studies were monocentric.

**Table 1:** Characteristics of studies included for meta-analysis

Trial	Design	Cancer	Enrollment size	Anti-CTLA-4	Dose (mg/kg)	CTC for AE version
Hodi [7]	RCT, phase III	Melanoma	676	Ipilimumab	3	3
Wilgenhof [14]	Prospective observational study	Melanoma	50	Ipilimumab	3	4
Delyon [15]	Prospective observational study	Melanoma	96	Ipilimumab	3	4
Margolin [16]	Open label, phase II	Melanoma	72	Ipilimumab	10	3
Hamid [17]	Randomized, double blind, phase II	Melanoma	82	Ipilimumab	3; 10	n/a
Danielli [18]	Single arm, phase II	Melanoma	13	Ipilimumab	10	3
Wolchok [19]	Randomised phase II, dose ranging study	Melanoma	217	Ipilimumab	0.3; 3; 10	3
O’Day [20]	Multicenter, single arm, phase II	Melanoma	155	Ipilimumab	10	3
Hersh [21]	RCT, phase II	Melanoma	74	Ipilimumab	3	2
Weber [22]	Randomized, double blind, phase II	Melanoma	115	Ipilimumab	10	3
Yang [23]	Randomized, double blind, phase II, dose ranging study	Renal cell	61	Ipilimumab	1; 3	n/a
Downey [24]	Multicenter, single arm, phase II	Melanoma	139	Ipilimumab	9	n/a
Ku [25]	Compassionate use trial	Melanoma	53	Ipilimumab	10	3
Di Giacomo [26]	Single arm, phase II	Melanoma	27	Ipilimumab	10	3
Royal [27]	Single arm, phase II	Pancreatic	27	Ipilimumab	3	3
Le DT [28]	Randomized, open label, phase IB	Pancreatic	30	Ipilimumab	10	3
Weber [29]	Phase I/II	Melanoma	88	Ipilimumab	n/a	n/a
Slovin [30]	Non randomized, open label, multicenter, phase I/II	Prostate	71	Ipilimumab	3; 5; 10	3
Calabro [31]	Open label, single arm, phase II	Mesothelioma	29	Tremelimumab	15	3
Chung [32]	Multicenter, single arm, phase II	Colorectal	47	Tremelimumab	15	3
Ralph [33]	Single arm, phase II	Gastric and esophageal	18	Tremelimumab	15	2
Ribas [34]	Phase I	Melanoma, renal cell, colon	39	Tremelimumab	10; 15	2

CTC for AE version, Common Terminology Criteria for Adverse Events version; RCT, Research clinical trial; n/a, Non-available

Anti-CTLA-4 antibodies were mainly given for melanomas. Other studies concerned renal cell carcinoma, mesothelioma and pancreatic, gastric, esophageal, colorectal, prostatic, and bladder cancer. The median duration of follow-up in these clinical trials was 23 months (IQR, 19–32). It is noteworthy to consider that, in 20 studies (90 %), patients with pre-existing autoimmune disease were not eligible for inclusion.

### Global incidence of irAEs

**Fig. 3:**
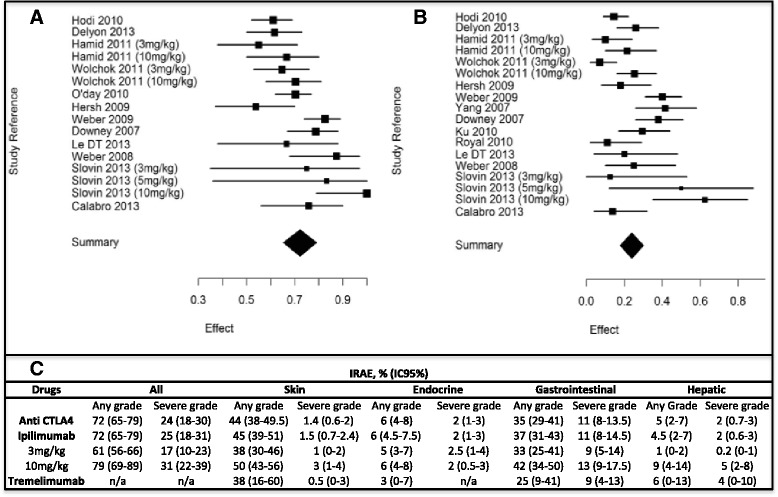
Incidence of global immune-related adverse events (irAEs) with anti-CTLA-4, all-grade (**a**) and severe grade (**b**). For ipilimumab treatment, different dosages were used: 3 mg/kg, 10 mg/kg, and 15 mg/kg. Only one study [31] reported global irAEs with tremelimumab treatment, at 15 mg/kg dosage. IrAEs associated with anti-CTAL-4 antibodies (**c**)

The overall incidence level of irAEs reported with anti-CTLA-4 treatment was 72 % (95 % CI, 65–79; I^2^, 81.94) for all-grade and 24 % (95 % CI, 18–30; I^2^, 79.97) for high-grade (Fig. 3a,b).

**Fig. 4:**
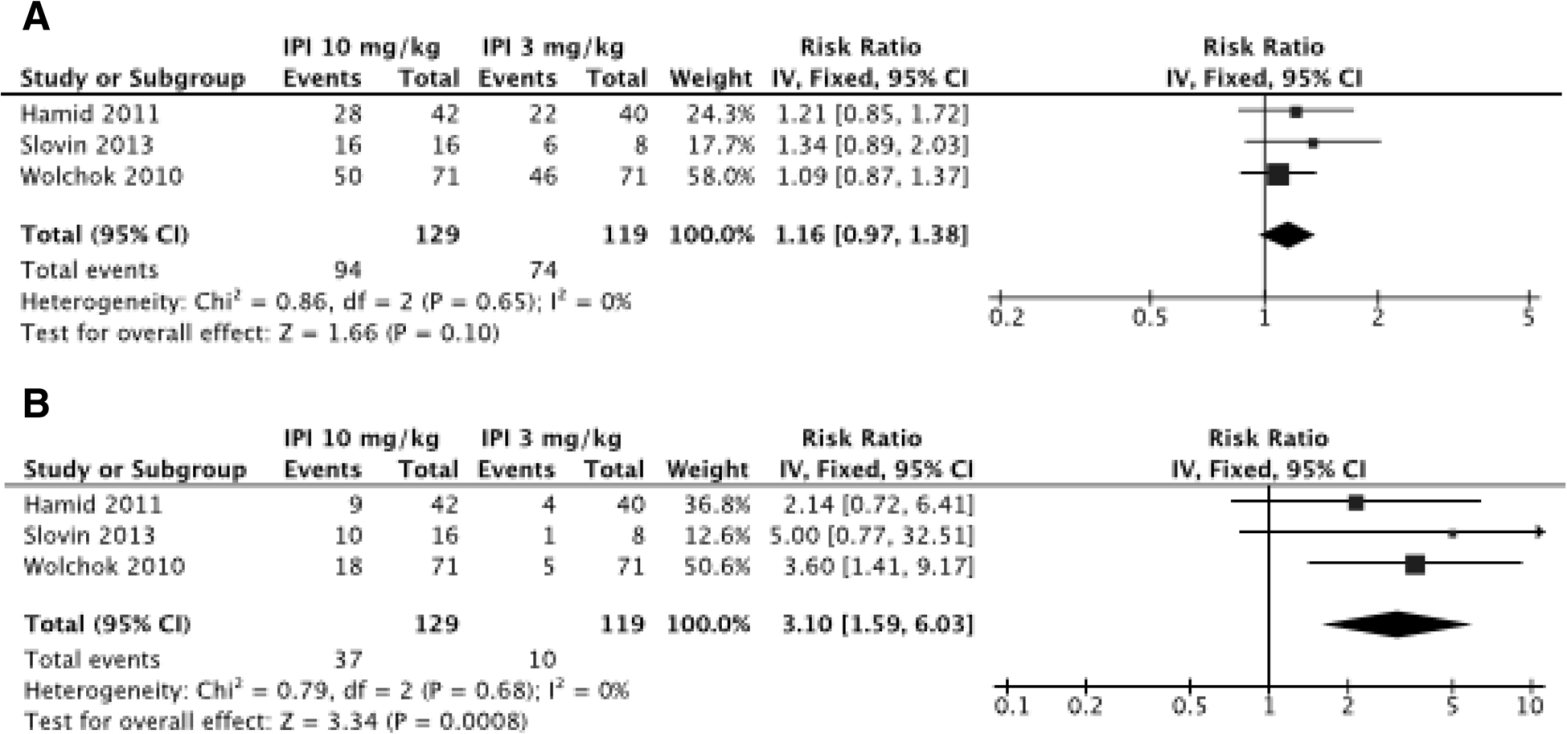
Risk ratio of developing an irAE with ipilimumab at 10 mg/kg compared with 3 mg/kg for global irAEs all grade (**a**) and high grade (**b**)

The incidence of all-grade irAEs varied according to the dosage of the drug, from 61 % (95 % CI, 56–66; I^2^, 0) in patients receiving ipilimumab at 3 mg/kg to 79 % (95 % CI, 69–89; I^2^, 85) in patients treated with ipilimumab 10 mg/kg. This dose effect was corroborated in high-grade irAEs, evaluated to 17 % (95 % CI, 10–23; I^2^, 71, 85) with ipilimumab 3 mg/kg and 31 % (95 % CI, 22–39; I^2^, 62) with ipilimumab 10 mg/kg (Figures S5, S6 and S7 in Additional file 1). To perform a statistical comparison between the two main doses (3 mg/kg and 10 mg/kg) we made a subgroup analysis within the three studies comparing the two doses [17, 19, 30]. The risk ratio (RR) of developing an irAE with ipilimumab at 10 mg/kg compared with 3 mg/kg was 3.10 (1.59–6.03; *P*=0.0008) for the overall incidence level of irAEs for high grade. RR of overall incidence level of irAEs for all grade did not reach a statistically significant difference (RR, 1.16 (0.97–1.38); *P*=0.10; Fig. 4a,b).

### Incidence of organ-specific irAEs

The skin and the gastrointestinal tract were mostly affected, in 44 % (95 % CI, 38–49.5) and 35 % (95 % CI, 29–41) of cases, respectively, while endocrine and hepatic organs were less affected, in 6 % (95 % CI, 4–8) and 5 % (95 % CI, 2–7), respectively. Other events, such as neurologic, hematologic, ophthalmologic, or rheumatologic diseases, were rare.

Almost all skin, endocrine, and hepatic irAEs were low grade (less than 5 %); high-grade irAEs remained more frequent in gastrointestinal events at 11 % (95 % CI, 8–13.5; Fig. 3c and Figure S8 to Figure S27 in Additional file 1).

As global irAEs, organ-specific irAEs seemed to be more important for patients treated with ipilimumab at 10 mg/kg except for high-grade endocrine irAEs (Fig. 3c). However, in the subgroup analysis [17, 19, 30], a statistically significant difference was only found between the two doses for all grade gastrointestinal irAEs with a RR of 1.43 (1.04–1.96; *P*=0.03; Figures S28 to S31 in Additional file 1).

There appeared to be fewer irAEs with tremelimumab, except those concerning hepatic disease.

### Incidence of death related to irAEs

Death due to irAEs occurred in 11 patients (0.86 %), often related to colic bowel perforation for patients with colitis.

### Nature of irAEs – data from case reports and retrospective studies

#### General characteristics

In addition to retrospective studies and some descriptive clinical trials, our research identified 100 patients from 57 case reports with at least one irAE [26, 36–91]. Among them, 18 presented several irAEs. The general characteristics of these patients are summarized in Additional file 1: Table S2. Anti-CTLA-4 treatments were mainly used in melanoma (94 %). Ninety-nine patients received ipilimumab and one patient received tremelimumab; 48 patients (48 %) had failed at least one previous oncologic therapy before receiving anti-CTLA-4 antibodies. The irAEs occurred in a median of 10 weeks (IQR, 6–12), within the first three cycles of ipilimumab treatment.

Interestingly, clinical remission (partial or complete), or at least cancer stabilization, was noted for 60 % of patients who experienced an irAE. When irAEs were diagnosed, anti-CTLA-4 was stopped in 76.2 % of patients.

#### Nature of irAEs

Cutaneous irAEs were by far the most common immune side effect of anti-CTLA-4 treatment and occurred within the first month of treatment. Pink red papules coalescing into thin plaques associated with mild to severe pruritus were usually described. A Koebner phenomenon was sometimes associated. This dermatitis was usually well tolerated and limited. It was mainly localized at proximal extensor surfaces of the limbs, trunk, and distal extremities. Palms and soles were often spared and head involvement was rare [92].

Histologically, an epidermal spongiosis and a superficial perivascular CD4 predominant T-cell infiltrate were present. An increased tissue (in papillary dermis) and peripheral blood eosinophil levels were described [93]. Treatment consisted of topical corticosteroids. Severe grades were treated with oral corticosteroids (starting at 1 mg/kg) and discontinuation of anti-CTLA-4 treatment.

Exacerbation of pre-existing dermatitis as eczema, vitiligo, or rosacea and extensive alopecia were also reported [36, 37, 92]. Moreover, a case of Sweet’s syndrome was reported in a patient undergoing ipilimumab therapy for metastatic melanoma [38]. After the second infusion, she developed fever and cutaneous eruption on her hands. Punch biopsy confirmed neutrophilic dermatitis and symptoms improved with corticosteroids therapy.

Endocrine irAEs were part of this spectrum of specific irAEs reported to anti-CTA-4 antibodies (Additional file 1: Table S3). They occurred within an average of 11 weeks but were not dose dependent, unlike other irAEs.

Autoimmune hypophysitis was the most frequent endocrine side effect, reported in up to 13 % of clinical trials. It was similar to lymphocytic hypophysitis, sharing the same clinical, biological, and radiological features [94]; 33 cases were reported. Symptoms were related to the anterior hypopituitarism (hormonal deficiencies) and pituitary mass effect [39]. Most patients presented headache (51.8 %: 14/27 patients), asthenia (59.3 %: 16/27), erectile dysfunction, and decreased libido. Visual disturbances were rare, reported only in one patient [40]. Indeed, mass syndrome was usually moderate: 3.4–6 mm in pre-treatment and 7.7–11.8 mm after ipilimumab therapy [39]. Affected areas were mainly the hypothalamic-pituitary-adrenal axis (92.9 %; 26/28 patients) and the thyrotropin axis (89.3 %; 25/28), followed by the gonadal axis (71.4 %; 20/28). The lactotrope and somatotropic axes were less frequently involved (21.4 % (6/28) and 10.7 % (3/28), respectively). One case of hypernatremia secondary to syndrome of inappropriate antidiuretic hormone and one case of diabetes insipidus were associated with ipilimumab-induced hypophysitis [41, 42]. MRI aided in the diagnosis of hypophysitis by identifying enlargements of the pituitary gland (68.2 %; 15/22) and homogeneous enhancements after injection of gadolinium (31.8 %; 7/22); MRI could be normal (6/22), without excluding the diagnosis. Treatment consisted of hormone substitution and high-dose oral corticosteroids with a median dose of 80 mg prednisone equivalent (IQR, 60–106), decreasing over 1 month and backed by hydrocortisone. MRIs normalized with corticosteroids in six patients. However, hormonal deficits persisted in 75 % (15/20) and required long-term hormonal replacement, especially corticosteroids. Five cases reported complete healing.

Hypo- and hyperthyroidism secondary to thyroiditis were rare, up to 5.6 % in clinical trials. One case reported bilateral Graves’ ophtalmopathy with high thyroperoxydase and thyroglobulin antibodies after two cycles of ipilimumab therapy [43].

Gastrointestinal irAEs were important and potentially severe immune complications reported with CTLA-4 blocking drugs (Additional file 1: Table S3). Patients received an average of three infusions (IQR, 2–4) before the onset of symptoms.

Colitis was reported in 21 patients. Clinical manifestations were diarrhea (95.2 %; 20/21), abdominal pain (38.1 %; 8/21), rectal blood (23.8 %; 5/21), and nausea, with or without fever. Colitis could be life threatening with fatal colic bowel perforation, reported in two patients [54].

Colonoscopy could be normal or reveal variable abnormalities, including edema, erythema, ulcers, friability, exudate, erosions, or bleeding [55].

Histology showed ulcerative epithelial defect (35.3 %; 6/17), lymphocytic (64.7 %; 11/17) or neutrophilic infiltrates (52.9 %; 9/17), or both (35.3 %; 6/17), and sometimes with eosinophil cells. Crypt micro-abscesses and architectural distortion were also occasionally seen [55]. Histopathology shared some features with inflammatory bowel disease and graft versus host disease. Colitis management is proposed in analogy with the treatment of these two pathologies [95].

Treatment consisted of symptomatic drugs (loperamide) and rehydration for grade 1 irAEs. For grade 2, anti-CTLA-4 discontinuation and moderate dose of oral steroids or budesonide are recommended. For severe grades, a high dose of steroids (sometimes intravenous) was recommended as first line treatment. In case reports, the median dose was 156 mg prednisone equivalent (IQR, 150–225) and in the event of no improvement in 1 week, infliximab (one or two infusions at 5 mg/kg) was proposed. For refractory colitis, a colectomy had to be performed [57]. Healing was reported in 91.5 % of patients.

Hepatitis was described in up to 19 % of clinical trials. Patients presented elevated alanine aminotransferase and aspartate aminotransferase, with or without hyperbilirubinemia, usually in the absence of clinical symptoms [63]. Biopsies from patients experiencing acute immune-related hepatotoxicity showed T-cell infiltrates (87.5 %; 7/8). Most patients responded to corticosteroids but several cases needed immunosuppressive therapies such as tacrolimus, mycophenolate, or antithymocyte globulin therapy [63, 64].

One case illustrated ipilimumab-related pancreatitis considered to be immune-related due to detection of anti-pancreas antibodies [66].

A case of coeliac disease was also reported [67]. Biology revealed anti-tissue transglutaminase and anti-gliadine antibodies. The duodenal biopsies showed a malabsorptive pattern; a gluten-free diet helped to stop diarrhea.

### Other irAEs

Various neurologic irAEs, such as Guillain-Barré syndrome [42, 68, 69], transverse myelitis [70], aseptic meningitis [42], inflammatory myopathy [71], orbital myositis [72], or myasthenia [70], were described (Additional file 1: Table S3). Despite adapted treatments (steroids, immunoglobulin therapy, and plamapheresis), authors reported three deaths and five patients with long-lasting neurologic irAEs related to anti-CTLA-4.

Additionally, six cases of sarcoidosis were reported [77–82]. Symptoms occurred in the first four cycles (IQR, 3–5) corresponding within the first 15 weeks (IQR, 12–24). Dyspnea (50 %; 3/6) and skin lesions (50 %; 3/6) were usually described. Computed tomography showed mediastinal lymph nodes in 83.3 % (5/6) of cases and micronodular and reticulonodular lesions in 80 % (4/6). Diagnosis was confirmed by biopsies (bronchial or cutaneous) revealing granulomas in all (5/5) cases. Healing was reported in all cases with oral steroids treatment at a dosage of 75 mg prednisone equivalent (IQR, 50–100).

Many other events were also reported with anti-CTLA-4 antibodies, including uveitis [83], organizing pneumonia [84], lupus nephritis [85], autoimmune cytopenia [85–87], and hemophilia A [89], as well as a recently published case of polymyalgia/giant cell arteritis [90].

### Quality assessment

Most of the studies were not blind or single-arm since the majority were compassionate trials. Thus, since investigators were aware of which treatment patients had received and of the possible drug side effects, it is possible that irAEs were over-reported. Despite these elements, most information was retrieved from trials with a moderate risk of bias. Further details regarding the quality assessment are available in Additional file 1: Table S4. No significant publication bias was found for all meta-analysis.

## Discussion

To our knowledge, this is the first systematic review and meta-analysis reporting overall published irAEs related to anti-CTLA-4. From 22 clinical trials included in the pooled analysis, we found a respective incidence of 72 % (95 % CI, 65–79; I^2^, 81.94) for all-grade irAEs and 24 % (95 % CI, 18–30; I^2^, 79.97) for high-grade irAEs leading to hospitalization or intravenous treatment. These results highlight the high risk of irAEs with anti-CTLA-4 drugs in patients with such metastatic cancers. These values were found to be quite similar to those of a retrospective review of safety data including 1498 patients treated with ipilimumab at various doses on 14 completed phase I–III trials [96], reporting inflammation drug-related adverse events in 64 %, with 18 % being of severe grades.

Among the wide spectrum of irAEs, cutaneous reactions and gastrointestinal tract immune side effects were commonly encountered, followed by endocrinopathies and hepatitis. However, several reports included nervous system disorders, sarcoidosis, respiratory, renal, or other organ immune involvement. Indeed, physicians of various specialties should be concerned by these irAEs and must examine all symptoms as being potentially induced by anti-CTLA-4 treatment. Voskens et al. [97] summarized and described, by organ system, the rare ipilimumab-induced immune side effects among 19 skin cancer centers. Through this complete description of previously unreported irAEs, several specialized centers shared their experience of irAEs in order to increase awareness and introduce an earlier management of potentially severe side effects. Recently, Teply et al. [98] also provided identification, description, and management of toxicities from immune checkpoint-blocking drugs (CTLA-4, PD-1, and PD-L1 antibodies).

An important clinical point is the early onset of irAEs, in general within the first 10 weeks, corresponding to a mean of three cycles for ipilimumab treatment, but varies according to the organ system involved. McDermott et al. [99] reported the occurrence of new ipilimumab-related immune toxicity in 7 of 78 patients with 2 years’ survival after ipilimumab treatment. These irAEs were mostly low grade and occurred more than 70 days after the last dose of ipilimumab. Three studies provide additional information on the safety profile of ipilimumab retreatment, comparable with that observed during ipilimumab induction and without new types of toxicity [100–102]. Lebbé et al. [100] observed a lower incidence of irAEs among patients retreated with ipilimumab 10 mg/kg who were previously treated with ipilimumab 3 mg/kg (67.6 %) and 10 mg/kg (56.6 %) compared with patients who received ipilimumab 0.3 mg/kg (75 %); this was also reported by Chiarion-Sileni et al. [101] in the Italian expanded program. They suggested the possibility of a dose-dependent CTLA-4 binding in caution with the selection bias since patients with high-grade irAEs in the parent study would not have been eligible for retreatment.

Few data are published on the long-term follow-up of anti-CTLA-4-induced irAEs. Indeed, anti-CTLA-4 antibodies are a recent approach in oncology and the follow-up in clinical trials is currently too short to assess long-term evolution of these side effects. Based on case reports, 91.5 % of patients experiencing a gastrointestinal (colitis) irAE and 100 % of patients with sarcoidosis recovered completely of their irAEs. However, patients with endocrine side effects (hypophysitis) were reported as healed in only 25 % of cases. This data is relevant with a recent study following up anti-CTLA-4-induced hypophysitis for over 2.5 years and reporting a long-term hormonal replacement requirement in 86.6 % of patients [103]. Indeed, the clinical features, management, and evolution of irAEs seem to be similar to known autoimmune diseases. These similarities beg the following questions: Do patients developing irAEs have particular genetic risk factors (e.g. HLA-DR alleles) such as those in autoimmune diseases? Could the CTLA-4 blockade be the trigger of long-term auto-immunity? Further studies are needed to provide these answers.

The overall incidence of irAEs observed with the two main doses of ipilimumab (3 mg/kg and 10 mg/kg) seem to be significantly different and have a dose-dependent effect since the CIs of means did not overlap. The subgroup analysis confirmed this hypothesis, with a RR at 3.10 (1.59–6.03; *P*=0.0008) of developing irAEs with a higher dosage (10 mg/kg) for high-grade global irAEs. It seems that treatment with tremelimumab has less irAEs, but the mechanism remains unclear. Patients receiving a lower dosage of FDA-approved therapy were not included in this meta-analysis, but it is noteworthy that there were very few irAEs for these sub-therapeutic doses [13].

Efficacy is also reported as having a dose-dependent effect [19]. A major question is the existence of a relationship between oncologic response and occurrence of irAEs, which could be indicative of tumor-specific T-cell activation. The present study has not been established to answer this question. However, based on case reports, irAE occurrence seems to be associated with clinical response to CTLA-4 blocking: 60 % of the patients presenting with irAEs experienced clinical remission (partial or complete) or at least cancer stabilization. Interestingly, several studies have also reported a strong correlation between clinical response to anti-CTLA-4 therapy and irAEs [23, 24, 95, 104, 105]. Thus, objective tumor response rates were around 30 % in patients who developed autoimmune events, while 0–10 % of the other patients responded to treatment. In a study by Downey et al. [24], all complete responders experienced high-grade irAEs. These observations corroborate the idea of a coupling autoimmunity and tumor immunity.

Under normal physiological conditions, CTLA-4 acts as a negative T-cell co-stimulatory signal, maintaining the peripheral T-cell homeostasis and tolerance to self or environmental antigens [106]. Expression on activated conventional T-cells is induced after T-cell receptor signaling (Fig. 1) while it is constitutively expressed on T-regulator cells (CD4+FoxP3+). CTLA-4 is now established as a critical regulator of T-regulator homeostasis and function [107]. Thus, if the CTLA-4 blockade enhances the intratumoral T-effector/T-regulator cell ratio in cancer patients, by depleting T-regulator cells [108], immunotherapy modulating CTLA-4 (such as abatacept) improves the regulatory T-cell inhibitory function in rheumatoid arthritis patients [109]. This example highlights the opposite therapeutic strategy between cancer patients and those affected by inflammatory/autoimmune diseases. Furthermore, T-regulator cell differentiation in the gut by commensal bacteria is known to actively engender mucosal tolerance. The frequency of colitis in patients receiving CTLA-4 blockade could be explained, in part, by the T-regulator depletion induced by this treatment [110].

Thus, given the critical role of CTLA-4 in immunologic homeostasis and the known irAE profile, patients with underlying autoimmune disease were usually excluded from clinical trials involving CTLA-4 blocking drugs. Few reports have been published concerning patients treated with ipilimumab despite an active autoimmune disease as rheumatoid arthritis, multiple sclerosis, ulcerative colitis, or Behçet’s disease [111, 112]. In three patients who presented rheumatoid arthritis, ulcerative colitis, and Behçet’s disease, no aggravation of the immune disorder was noted and their cancer benefitted from the treatment. Although anecdotal, these descriptions raise the question of the current exclusion of anti-CTLA-4 treatment in patients with autoimmune disease, given the poor prognosis of a metastatic cancer. Furthermore, Lipson et al. [113] reported two successful administrations of ipilimumab to patients receiving an immunosuppressive regimen for kidney transplantation. It illustrates that ipilimumab could be a safe and effective option for solid organ transplantation in patients with a higher risk of melanoma.

The management of immune toxicities has been well-developed by the manufacturer by working with the FDA to edit and diffuse irAE management guidelines [114]. In parallel, experts detailed and considered the management of irAEs by organ system [98, 115, 116]. Patients usually required high-doses of corticosteroids according to the irAE type and grade. Of note, a prophylactic strategy with budesonide to prevent ipilimumab-induced colitis was ineffective, with a similar rate of diarrhea/colitis for patients receiving budesonide and those receiving placebo [22]. Another important clinical finding is the lack of evidence that steroid administration affects oncologic response [95, 104]; immunosuppressive agents were used in the event of no early improvement (e.g. infliximab in colitis).

Ongoing research focuses on the identification of predictive biomarkers of treatment response and of irAE occurrence. A limited proportion of patients receiving CTLA-4-blocking drugs achieve the objective tumor response, while most have irAEs. Individual data from these long-term survivors will help identify genetic and/or immunological biomarkers in order to define patient subsets likely to benefit from immunotherapy with adequate immune monitoring. Preliminary studies have investigated the genetic variation in CTLA-4; some variants seem to influence the response to therapy with improved overall survival and no occurrence of irAEs [117, 118]. Snyder et al. [119] also defined a genetic basis for benefit from CTLA-4 blockade in melanoma by whole-exome sequencing on tumors and matched blood samples. They identified a neo-antigen landscape specifically present in tumors with a strong response to CTLA-4. Regarding immunological parameters, a significant increase in T-cell ICOS expression (costimulatory molecule, third member of the CD28/CTLA-4 family) following ipilimumab treatment was found in melanoma patients who experienced disease control [120]. Recently, Vudattu et al. [121] described a reconstituted “humanized” mice model of human autoimmune disease *in vivo* that may provide insights into anti-CTLA-4 antibody effects on autoimmunity. These “humanized” mice treated with anti-CTLA-4 antibodies develop hepatitis, adrenalitis, and sialitis, as well as anti-nuclear antibodies (IgM or IgG). Thus, this model could be relevant to describe the irAEs observed in humans treated with anti-CTLA-4 antibodies and explore the immunologic pathways of these side effects [121].

Our study has some limitations. First, the diagnosis of irAEs may vary among investigators as definitions of irAEs in clinical trials are unclear. In Hodi et al. [7], an irAE was defined as an adverse event that was associated with exposure to the study drug and that was consistent with an immune phenomenon. For example, a rash could be a dermatologic irAE or an allergic reaction, and unfortunately we do not know if all patients with a rash were given a biopsy. This may lead to an overestimation of the incidence of irAEs associated with anti-CTLA-4. However, when the immune characteristic of the drug-related adverse event was not specified, we did not record it. It would have been better to present results with an odds ratio to evaluate a risk, but this was not possible because most oncologic studies are single arm or compared with a chemotherapy gold standard, not a placebo. We selected patients receiving anti-CTLA-4 antibodies alone, and not in combination treatment, in order to estimate the real incidence of irAEs induced by these molecules. Most studies and reports concerned ipilimumab treatment due to the marketing authorization in advanced melanoma and future data concerning tremelimumab and other immunotherapies will be interesting. Finally, high level of heterogeneity was observed in this meta-analysis (around 80 % for the majority of calculations). This heterogeneity was taken into account by performing random effects models, and its principal source was certainly the heterogeneity of the studies analyzed (differences in patient’s profiles, various dosages of treatments, etc.).

Given the “up-to-date” subject and the emergence of cancer immunotherapy, increasing reports of anti-CTLA-4-induced irAEs are published. Indeed, since our deadline of literature search, various auto-immune hematological [122, 123], renal [124], cutaneous [125–128], ophthalmologic [129–132], neurologic [133, 134], endocrine [132, 135–137], gastrointestinal [138, 139], and a central nervous system sarcoidosis [140] cases have been described.

## Conclusion

The potential price of a long-term cure of metastatic tumors is atypical immune toxicity, reflecting the immune mechanism of action of anti-CTLA-4 antibodies. A better knowledge of these irAEs and their management in a multidisciplinary approach will help to reduce morbidity and to guide therapy interruptions. Further studies are required to identify specific patient characteristics and/or biomarkers that may be associated with ipilimumab clinical efficacy in patients who did and did not develop an irAE.

## Additional file

Additional file 1:
**Figures S5 to S7 Global immune-related adverse events (irAEs) with ipilimumab all dosage, 3 mg/kg, and 10 mg/kg for all grades and high grade.** **Figures S8 to S27** Organ-specific irAEs (endocrine, skin, gastrointestinal, and hepatic) for ipilimumab all dosage, 3 mg/kg, and 10 mg/kg and Tremelimumab, for all grades and high grade. **Figures S28 to S31** Risk ratio of developing irAEs with ipilimumab at 10 mg/kg comparing with 3 mg/kg for organ-specific irAEs (gastrointestinal, skin, endocrine, and hepatic). **Table S2** General characteristics of patients receiving anti-CTLA4 antibodies described in case reports. **Table S3** Organ-specific irAEs. **Table S4** Quality assessment. (DOCX 12329 kb)

## Abbreviations

CTLA-4: Cytotoxic T-lymphocyte-associated antigen-4
FDA: Food and Drug Administration
irAEs: Immune-related adverse events
