# Supplementary material for: Immune related adverse events associated with anti-CTLA-4 antibodies: systematic review and meta-analysis
Source: BMC Med. 2015 Sep 4;13:211. doi: 10.1186/s12916-015-0455-8 (PMC4559965; doi:10.1186/s12916-015-0455-8)
Supplement: Additional file 1: — Figures S5 to S7 Global immune-related adverse events (irAEs) with ipilimumab all dosage, 3 mg/kg, and 10 mg/kg for all grades and high grade. Figures S8 to S27 Organ-specific irAEs (endocrine, skin, gastrointestinal, and hepatic) for ipilimumab all dosage, 3 mg/kg, and 10 mg/kg and Tremelimumab, for all grades and high grade. Figures S28 to S31 Risk ratio of developing irAEs with ipilimumab at 10 mg/kg comparing with 3 mg/kg for organ-specific irAEs (gastrointestinal, skin, endocrine, and hepatic). Table S2 General characteristics of patients receiving anti-CTLA4 antibodies described in case reports. Table S3 Organ-specific irAEs. Table S4 Quality assessment. (DOCX 12329 kb) [file 12916_2015_455_MOESM1_ESM.docx]

**Results**

**Meta-analysis**

1. All immune related adverse events
   1. Ipilimumab


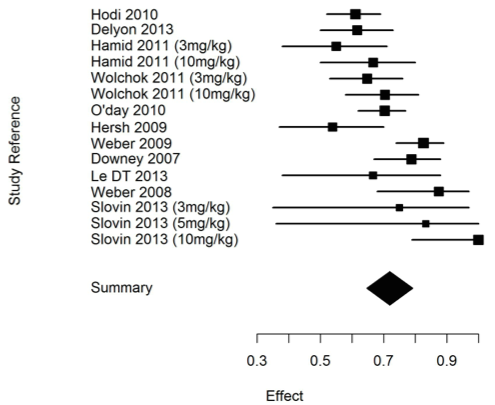

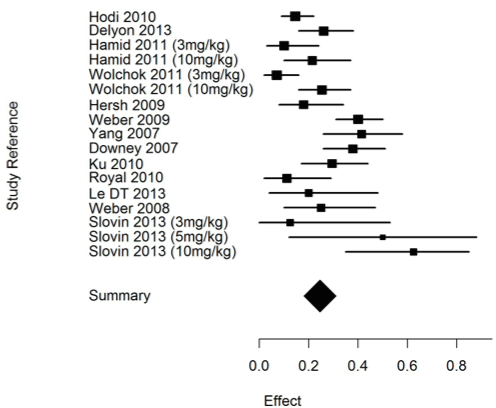


A

B

**Figure 5** - Incidence of global irAEs with ipilimumab all dosage (3mg/kg, 10mg/kg, 5mg/kg), all-grade (**A**) and severe grade (**B**).


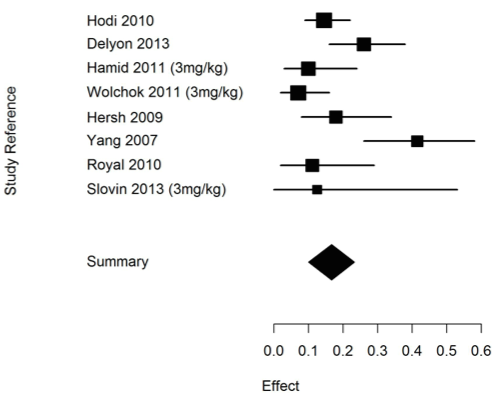

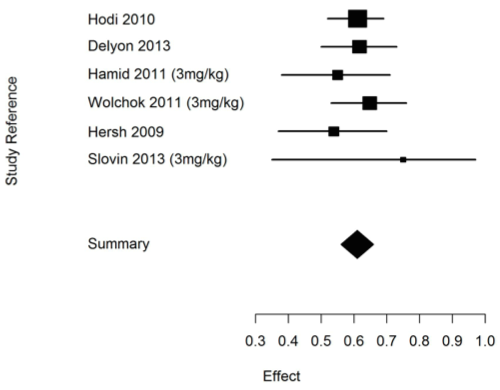


A

B

**Figure 6** - Incidence of global irAEs with ipilimumab 3mg/kg, all-grade (**A**) and severe grade (**B**).


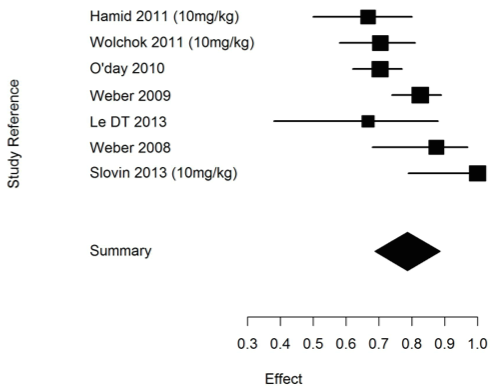

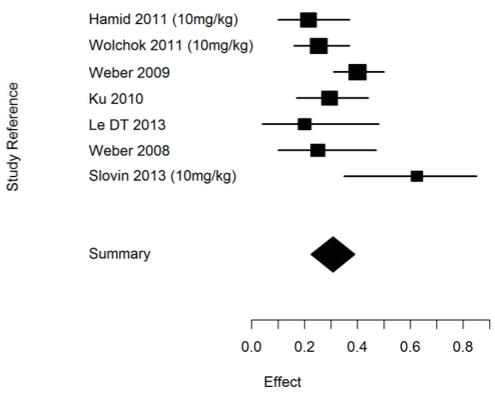


A

B

**Figure 7** - Incidence of global irAEs with ipilimumab 10mg/kg, all-grade (**A**) and severe grade (**B**).

- 1. Tremelimumab

Only one study (Calabrò *et al.* 2013) reported global irAEs with tremelimumab treatment, at 15mg/kg dosage. A meta analysis was not possible in this group.

1. Endocrinologic immune related adverse events
   1. Anti-CTLA-4


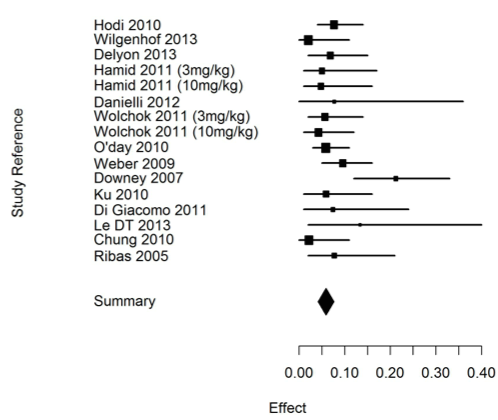

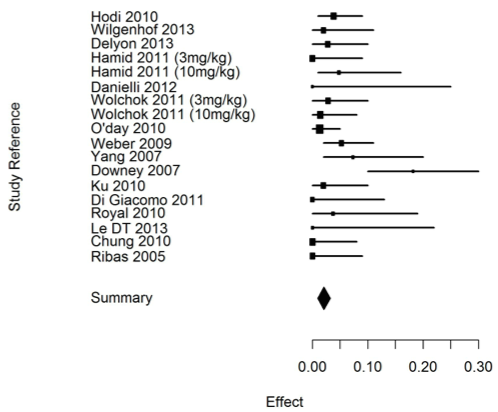


A

B

**Figure 8** - Incidence of endocrinologic irAEs with anti-CTLA-4, all-grade (**A**) and severe grade (**B**).

- 1. Ipilimumab


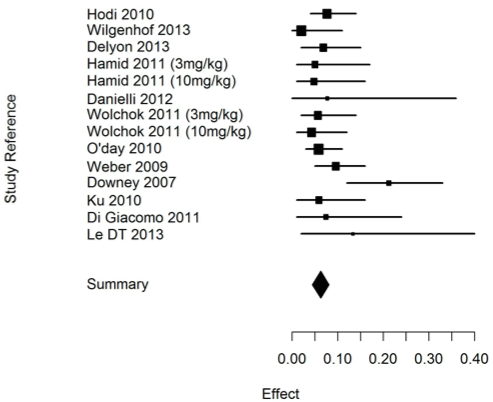

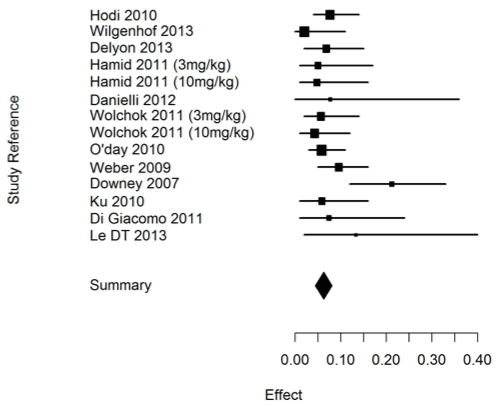


A

B

**Figure 9** - Incidence of endocrinologic irAEs with ipilimumab all dosage, all-grade (**A**) and severe grade (**B**).


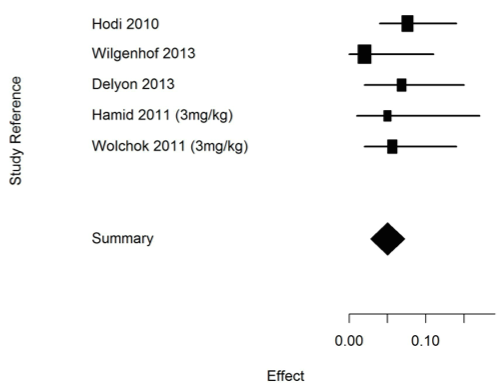

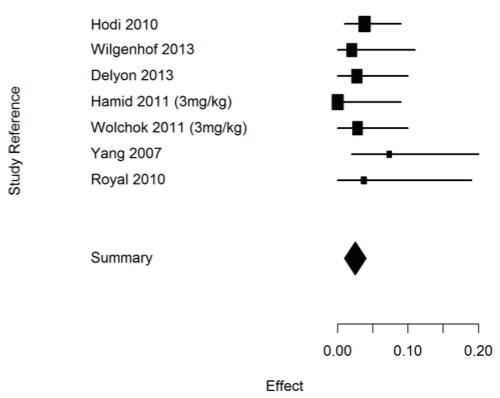


A

B

**Figure 10** - Incidence of endocrinologic irAEs with ipilimumab 3mg/kg, all-grade (**A**) and severe grade (**B**).


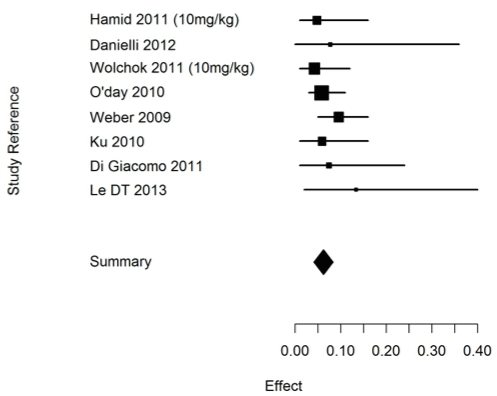

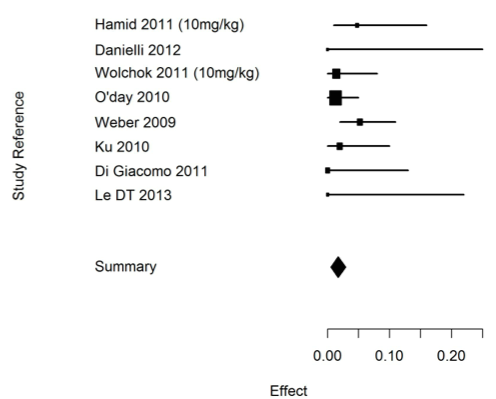


A

B

**Figure 11** - Incidence of endocrinologic irAEs with ipilimumab 10mg/kg, all-grade (**A**) and severe grade (**B**).

- 1. Tremelimumab


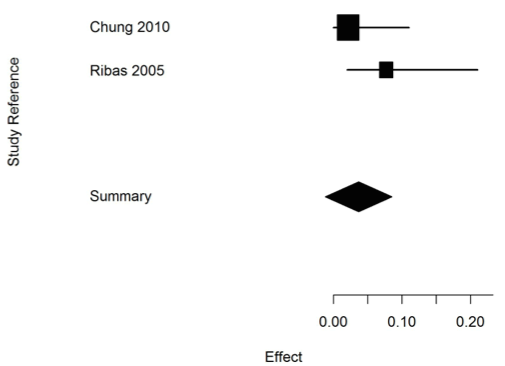


**Figure 12** - Incidence of endocrinologic irAEs with tremelimumab, all-grade. Meta analysis was not possible for severe grade.

1. Cutaneous immune related adverse events
   1. Anti CTLA4


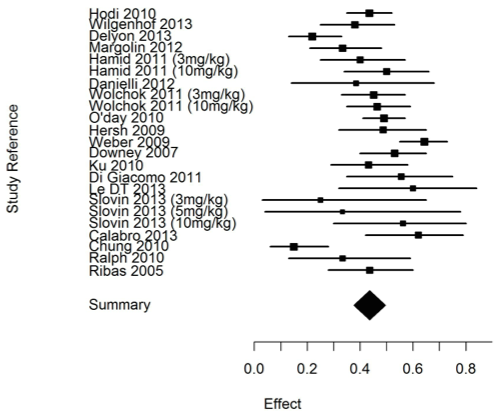

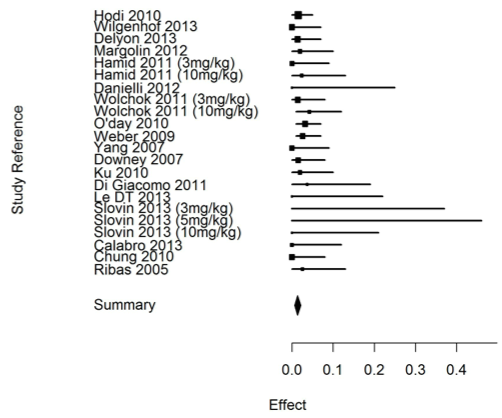


A

B

**Figure 13** - Incidence of cutaneous irAEs with anti-CTLA-4, all-grade (**A**) and severe grade (**B**).

- 1. Ipilimumab


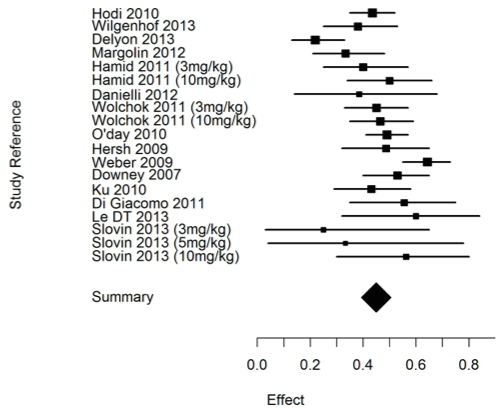

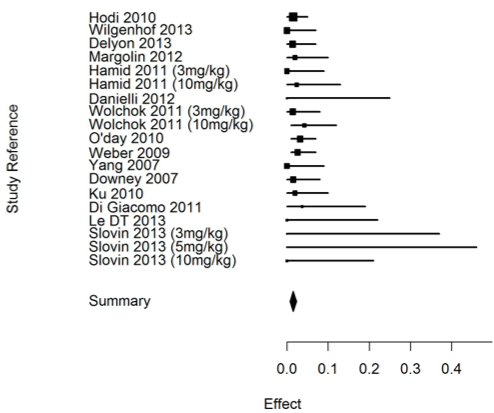


A

B

**Figure 14** - Incidence of cutaneous irAEs with ipilimumab all dosage, all-grade (**A**) and severe grade (**B**).


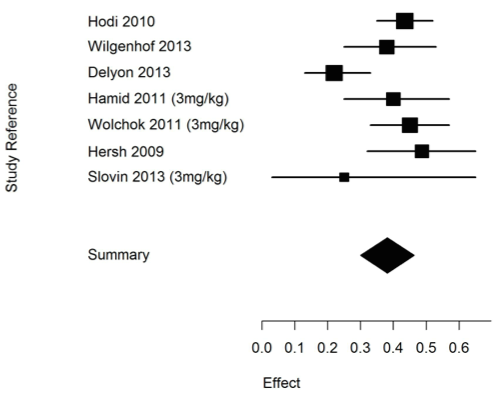

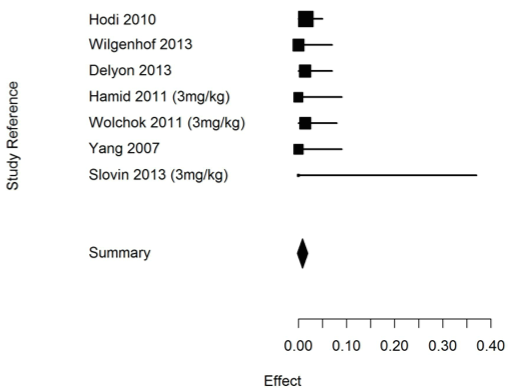


A

B

**Figure 15** - Incidence of cutaneous irAEs with ipilimumab 3mg/kg, all-grade (**A**) and severe grade (**B**).


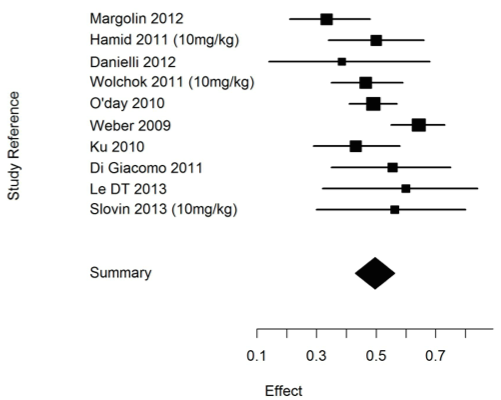

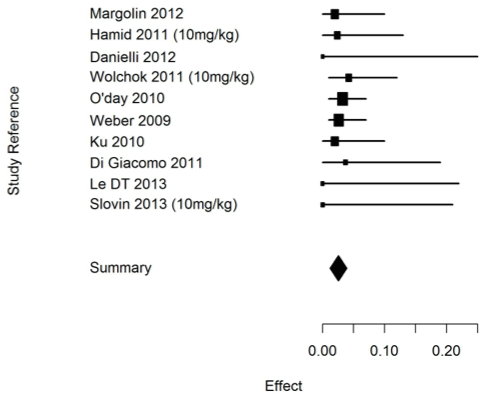


A

B

**Figure 16** - Incidence of cutaneous irAEs with ipilimumab 10mg/kg, all-grade (**A**) and severe grade (**B**).

- 1. Tremelimumab


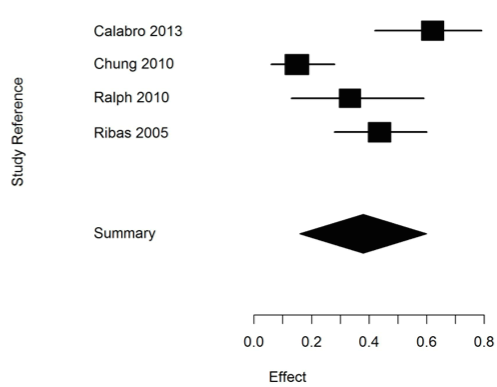

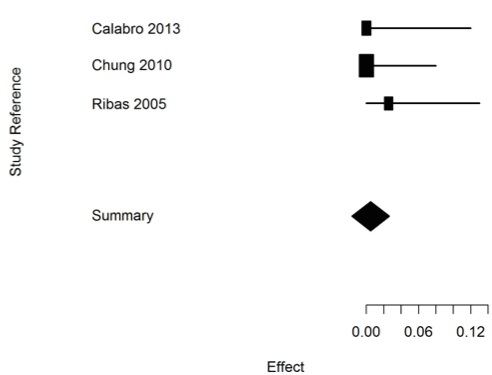


A

B

**Figure 17** - Incidence of cutaneous irAEs with tremelimumab, all-grade (**A**) and severe grade (**B**).

1. Gastro intestinal immune related adverse events
   1. Anti CTLA4


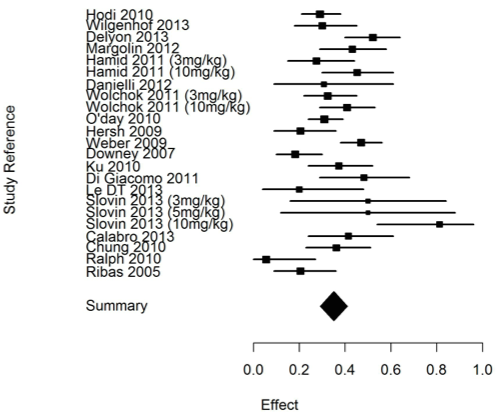

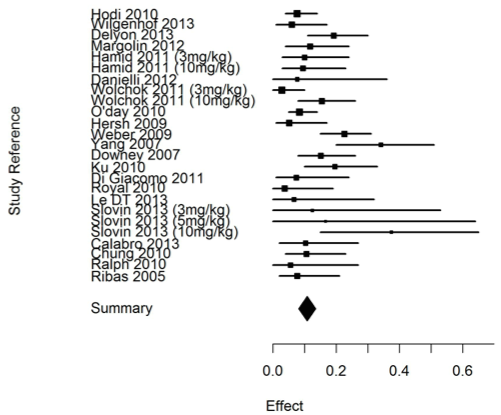


A

B

**Figure 18** - Incidence of gastro intestinal irAEs with anti CTLA4, all-grade (**A**) and severe grade (**B**).

- 1. Ipilimumab


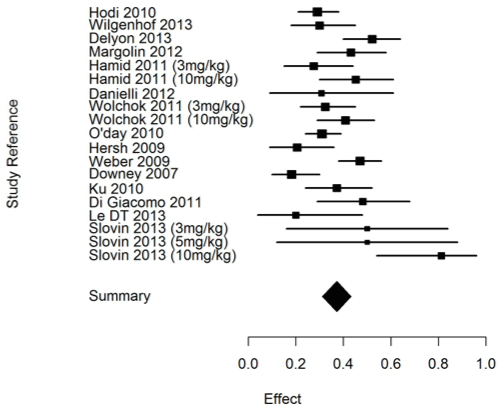

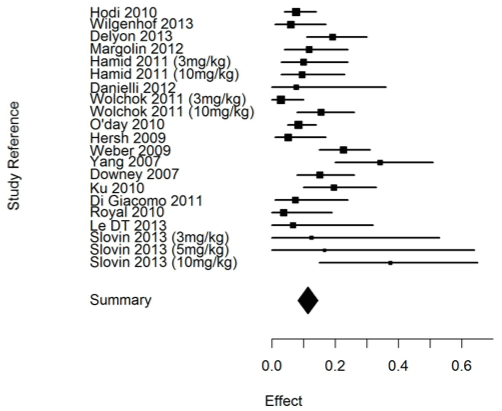


A

B

**Figure 19** - Incidence of gastro intestinal irAEs with ipilimumab all dosage, all-grade (**A**) and severe grade (**B**).


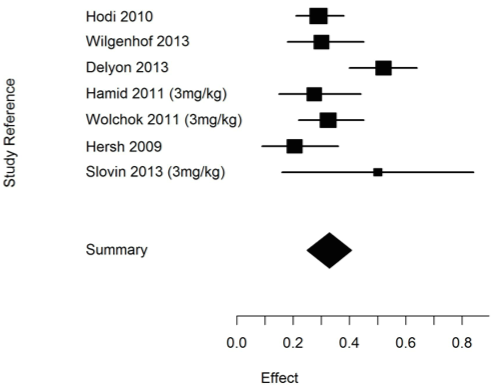

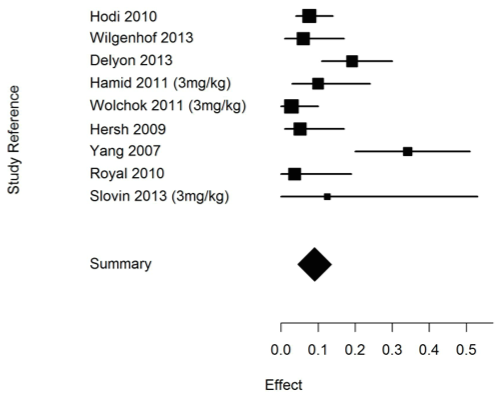


A

B

**Figure 20** - Incidence of gastro intestinal irAEs with ipilimumab 3mg/kg, all-grade (**A**) and severe grade (**B**).


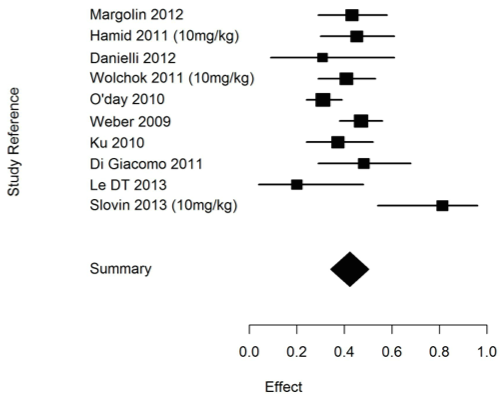

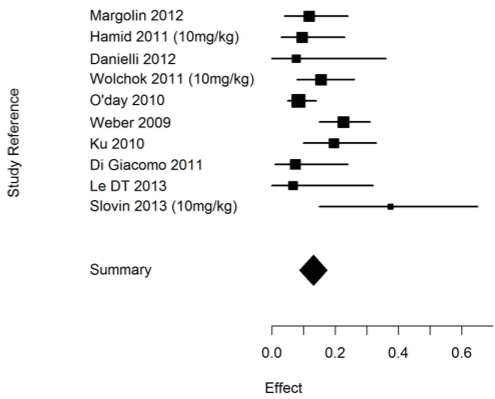


A

B

**Figure 21** - Incidence of gastro intestinal irAEs with ipilimumab 10mg/kg, all-grade (**A**) and severe grade (**B**).

- 1. Tremelimumab


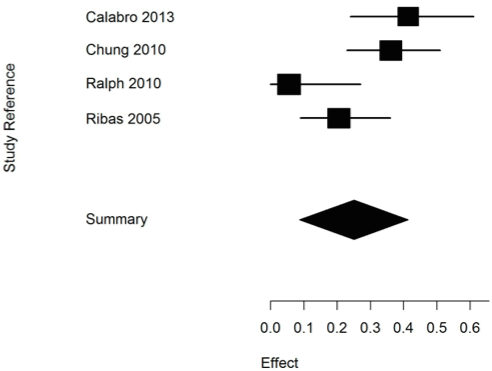

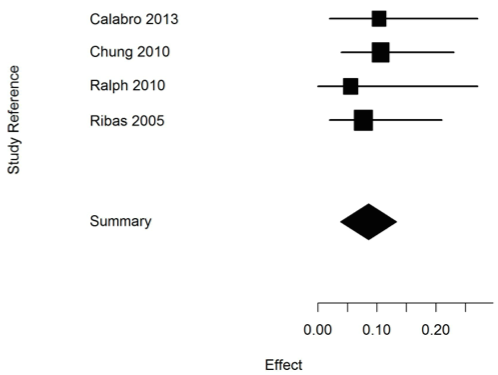


A

B

**Figure 22** - Incidence of gastro intestinal irAEs with tremelimumab, all-grade (**A**) and severe grade (**B**).

1. Hepatic immune related adverse events
   1. Anti-CTLA-4


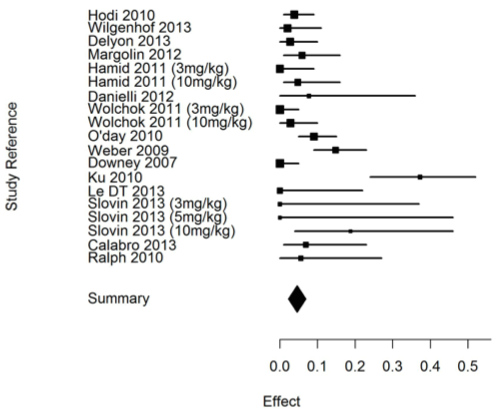

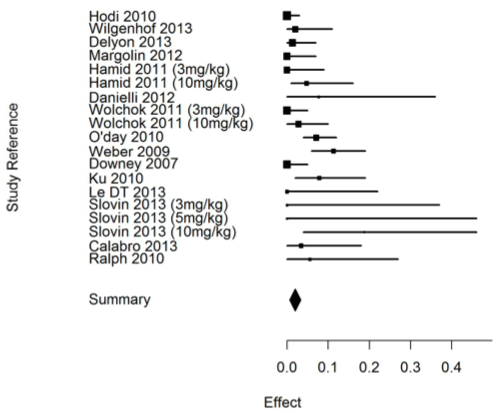


A

B

**Figure 23** - Incidence of gastro intestinal irAEs with anti-CTLA-4, all-grade (**A**) and severe grade (**B**).

- 1. Ipilimumab


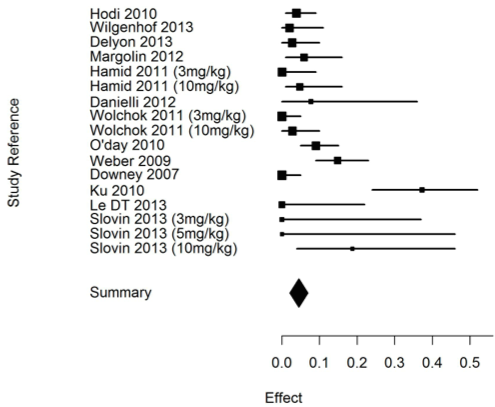

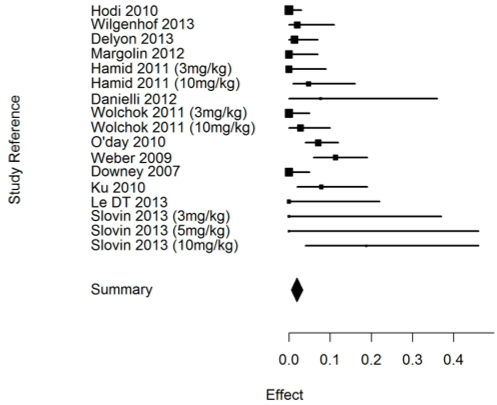


A

B

**Figure 24** - Incidence of hepatic irAEs with ipilimumab all dosage, all-grade (**A**) and severe grade (**B**).


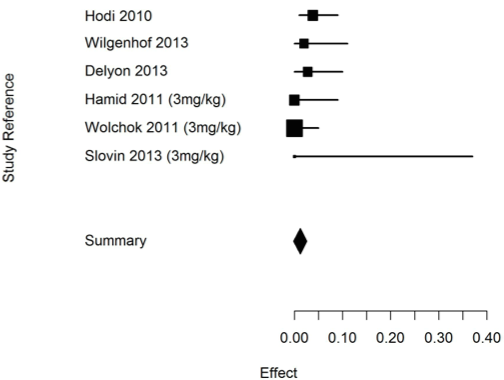

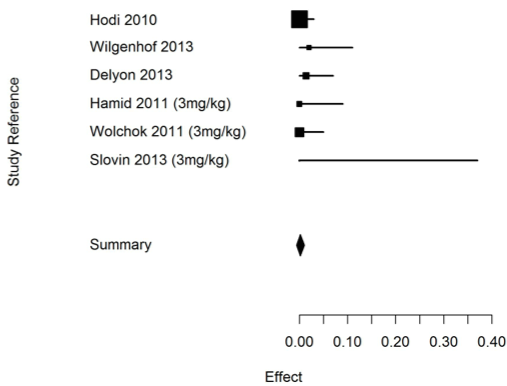


A

B

**Figure 25** - Incidence of hepatic irAEs with ipilimumab 3mg/kg, all-grade (**A**) and severe grade (**B**).


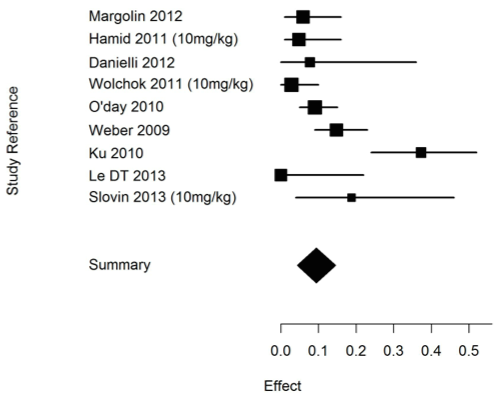

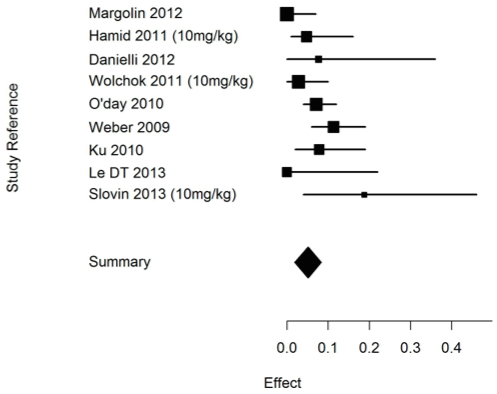


A

B

**Figure 26** - Incidence of hepatic irAEs with ipilimumab 10mg/kg, all-grade (**A**) and severe grade (**B**).

- 1. Tremelimumab


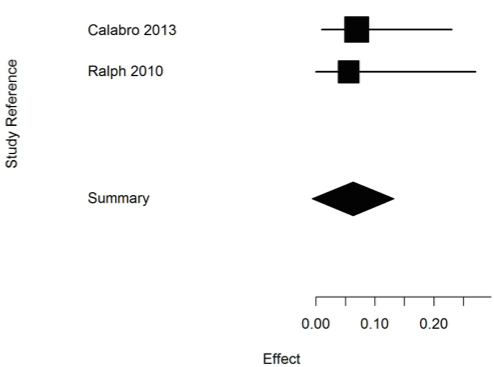

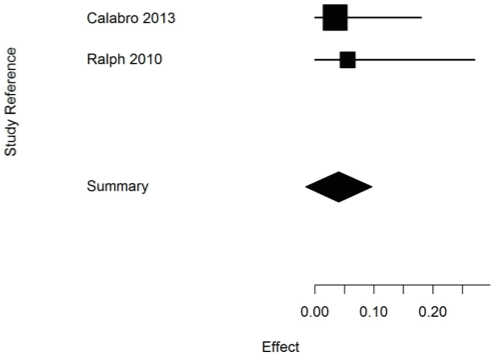


A

B

**Figure 27** - Incidence of hepatic irAEs with tremelimumab, all-grade (**A**) and severe grade (**B**).


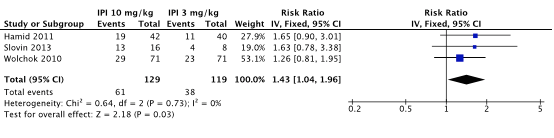

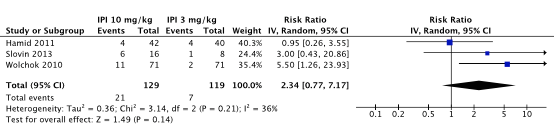


B

A

**Figure 28** –Risk ratio of developing a gastro intestinal irAE with ipilimumab at 10mg/kg comparing with 3mg/kg for gastro intestinal irAEs all grade (**A**) and high grade (**B**).


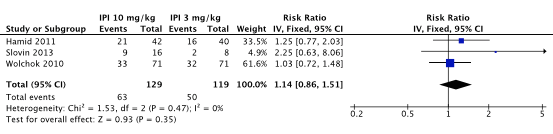

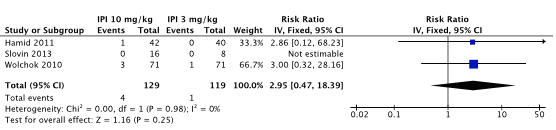


A

B

**Figure 29** - Risk ratio of developing a cutaneous irAE with ipilimumab at 10mg/kg comparing with 3mg/kg for cutaneous irAEs all grade (**A**) and high grade (**B**).


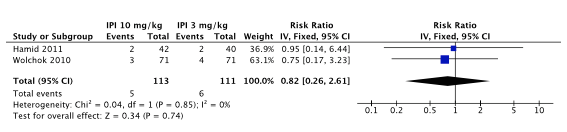

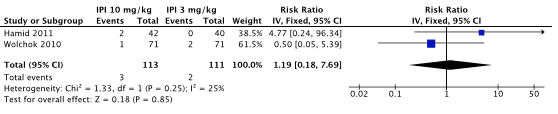


A

B

**Figure 30** – Risk ratio of developing an endocrine irAE with ipilimumab at 10mg/kg comparing with 3mg/kg for endocrine irAEs all grade (**A**) and high grade (**B**).


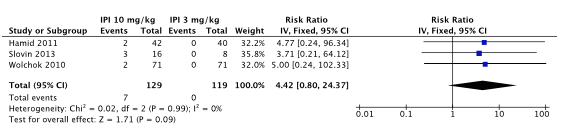


**Figure 31** - Risk ratio of developing a hepatic irAE with ipilimumab at 10mg/kg comparing with 3mg/kg for hepatic irAEs all grade (which are all high grade).

**Systematic review**

**Table 2** - General characteristics of patients receiving anti-CTLA-4 antibodies described in case reports

**Table 3** - Organ specific irAEs.

**Quality assessment**

**Table 4** - Quality assessment - + low risk of bias, - high risk of bias, ? uncertain risk of bias, n/a not applicable.
